# Improving an Industrial Sherry Base Wine by Yeast Enhancement Strategies

**DOI:** 10.3390/foods11081104

**Published:** 2022-04-12

**Authors:** Marina Ruiz-Muñoz, Gustavo Cordero-Bueso, Pedro Miguel Izquierdo-Cañas, Adela Mena-Morales, Jesús M. Cantoral

**Affiliations:** 1 Departamento de Biomedicina, Biotecnología y Salud Pública, Universidad de Cádiz, Avenida República Árabe Saharaui s/n, Puerto Real, 11510 Cadiz, Spain; marina.ruiz@uca.es (M.R.-M.); jesusmanuel.cantoral@uca.es (J.M.C.); 2 Instituto Regional de Investigación y Desarrollo Agroalimentario y Forestal de Castilla-La Mancha (IRIAF), IVICAM. Ctra. Albacete s/n, 13700 Tomelloso, Spain; pmizquierdo@jccm.es (P.M.I.-C.); amenam@jccm.es (A.M.-M.); 3 Parque Científico y Tecnológico de Castilla-La Mancha, Paseo de la Innovación 1, 02006 Albacete, Spain

**Keywords:** industrial wine yeasts, *S. cerevisiae*, mass-mating, adaptive evolution, volatile compounds, sensory analysis

## Abstract

There is growing interest in yeast selection for industrial fermentation applications since it is a factor that protects a wine’s identity. Although it is strenuous evaluating the oenological characteristics of yeasts in selection processes, in many cases the most riveting yeasts produce some undesirable organoleptic characteristics in wine. The aim of the present work is to improve an industrial yeast strain by reducing its hydrogen sulfide (H_2_S) production. To accomplish this, two different improvement approaches were used on said yeast: hybridization by mass mating and adaptive laboratory evolution, both performed through spore generation and conjugation, thus increasing genetic variability. Three evolved variants with lower H_2_S production were obtained and used as starters to carry out fermentation at an industrial level. Wine quality was analyzed by its principal oenological parameters and volatile aroma compounds, which were both corroborated by sensory evaluations. Significant differences between the produced wines have been obtained and a substantial improvement in aromatic quality has been achieved. Both hybrids were the most different to the control due to terpenes and esters production, while the evolved strain was very similar to the parental strain. Not only have organoleptic defects been reduced at an industrial level, more floral and fruitier wines have been produced.

## 1. Introduction

The production of Fino and Manzanilla wines from the Jerez-Xèrés-Sherry Appellation of Origin follows two successive processes: firstly, an alcoholic fermentation of must from *Vitis vinifera* var. Palomino Fino to obtain a “young” base white wine takes place. Then, wines to be aged biologically under a veil of flor are fortified up to 15% *v*/*v* ethanol content, if necessary; meanwhile, those destined for the production of Amontillado or Oloroso wines are fortified up to 17–18% *v*/*v* [1]. Palomino Fino is considered a low aromatic grape variety, but it offers a high yield in warm climates [2,3].

Sherry base wine fermentation has been traditionally carried out by *Saccharomyces cerevisiae* strains naturally present on grapes and winery equipment. However, climatic change is exerting an increasing influence on vine phenology, grape composition and (lately) fermentation performances, wine microbiology and chemistry, and sensory traits [4]. Thus, to overcome the possible inconveniences derived from increasing climatic change and to improve the organoleptic characteristics of the sherry base white wines, an increasing number of wineries use selected yeast strains as a starter culture. 

It is well known that during the alcoholic fermentation of sugars, yeast strains not only produce ethanol and carbon dioxide but also several flavour compounds that greatly influence the organoleptic characteristics of the final product [5]. Although a large number of commercial strains are available and prepared to be used as inocula, there is an increasing interest in the selection of autochthonous yeast strains for their use as a starter, since they produce differentiable and distinctive wines [6,7]. Despite the abundance of indigenous yeast diversity, the selective and specific conditions of industrial wine fermentations sometimes require phenotypic characteristics that cannot be easily found in nature [8]. Thus, it is possible to carry out genetic improvement programs to enhance the selection of wine yeast starters able to guarantee wine quality [9,10,11]. Considering the lack of acceptance for the use of genetic-modified organisms in the majority of the states in the world, techniques such as classical mutagenesis, adaptive evolution, cytoduction, and spheroplast fusion and hybridization are currently used to obtain novel yeast strains [9,11,12]. They have been extensively used in industrial antibiotic and enzyme production but not in winemaking [13]. However, non-GMO yeasts (without any genetic engineering) could show variations during industrial wine fermentation such as alterations in the stress-related response, fermentative performance and the chemical profile due to the action of secondary metabolites [14]. Several studies have shown the benefits and potential of the use of non-GMO yeast strains in winemaking at laboratory or semi-industrial scales [15,16]. Nevertheless, there is a gap in the literature on the efficacy of improved yeast strains with respect to large-scale or industrial vinification performance and their impact on aromatic profiles.

Besides their fermentation capability, yeast selection criteria are based on several attributes, including flavour characteristics and metabolic and technological properties [5]. The importance of these criteria depends on the type and style of wine to be made as well as the requirements of the winery [13]. High ethanol tolerance, low volatile acidity production, and the absence of off-flavours such as hydrogen sulfide are highly desirable properties for yeast selection by winemakers [17]. Although selection is an arduous process of evaluation of the oenological characteristics of yeasts, in many cases the most interesting yeasts possess some undesirable properties. While the desirability of some of the metabolites produced by yeasts during fermentation depends on many factors, including their concentration or wine style, others are usually considered contributors to off-flavour [18]. This is the case with hydrogen sulfide (H_2_S), which is produced during fermentation, by strains belonging mainly to the species *S. cerevisiae* [19]. H_2_S is associated with an undesirable rotten egg-like flavour and its presence is easily noticeable since its detection threshold is 50–80 µg/L in wine [20]. The amount of hydrogen sulfide produced depends on the yeast strain, the fermentation conditions, and the concentration of nutrients in the must, such as assimilable nitrogen or vitamins and the availability of sulphur compounds [21]. Most of the hydrogen sulfide produced during fermentation arises because of sulphur-containing amino acid biosynthesis (i.e., methionine and cysteine) through the sulphate reduction pathway (SRS). These amino acids are essential for the growth of *S. cerevisiae*, so yeast strains must synthesize them if they are not present in the must [9].

In this work, two different strategies based on sexual recombination were used in order to obtain non-genetically modified yeast strains with lower production of H_2_S from a diploid wine yeast strain that produces significant quantities of H_2_S during alcoholic fermentation. The yeast strains obtained were used as a starter culture of industrial-level fermentations and volatile compounds and sensory analyses of the sherry base wines obtained were carried out. 

## 2. Materials and Methods

### 2.1. Yeast Strains and Growth Conditions

The parental yeast strain used in this study to obtain evolved variants belonged to *S. cerevisiae* UCA-Y-001 (formerly P5). It was previously isolated from the Palomino Fino white grape variety, selected and characterized in our laboratory by Rodríguez et al. [6]. Since then, it has been used as an inoculum to carry out fermentation at an industrial level. Furthermore, in the hybridization procedure, another yeast parental strain was used, CLI-S (formerly S), a low acetic acid and H_2_S-producing *S. cerevisiae* strain previously isolated from the Malvar white grape variety, selected and characterized in the Madrid winemaking area [7]. 

The yeast strains were routinely cultured on YPD agar plates (2% glucose, 2% peptone, 1% yeast extract, 2% agar) and incubated at 28 °C for 24 h. For long-term preservation, all the strains were stored at −80 °C in cryotubes supplemented with glycerol (final concentration of 40%).

### 2.2. Sporulation

Parental yeasts were induced to sporulation using a protocol previously described [11] with some modifications. Yeast pre-cultures were developed in YPD medium at 28 °C over 3 days and then 100 μL was seeded in a sporulation medium (1% potassium acetate, 2% agar) and incubated at 25 °C. Spores were obtained using a standard Zymolyase protocol.

The total numbers of cells and spores were counted in a Neubauer^®^ chamber and the efficiency was calculated. Then, asci were dissected using a micromanipulator microscope Nikon Eclipse (Nikon, Tokyo, Japan), distributed on YPD-agar plates and incubated during 2–5 days at 28 °C. The viability of the spores was determined as the ability of a single colony to grow after tetrad dissection.

### 2.3. Generation of Intraspecific Hybrid Yeasts

#### 2.3.1. Determination of Phenotypic Markers from Parental Yeasts

A screening for complementary phenotypic markers of both parental strains UCA-Y-001 and CLI-S as well as their spores was carried out in order to distinguish potential hybrids from parental yeast strains. A 96-well test for ability to ferment or assimilate different carbon sources and for growth under different concentrations of SO_2_ (0, 150, 200, 250 mg/L), cycloheximide (0, 25, 50, 75, 100 mg/L), ethanol (5, 10, 11, 12, 14, 15% *v*/*v*), pH (from 2.5 to 3.5), and temperature (15, 17, 20, 15, 37 °C) was performed as described by [22].

#### 2.3.2. Mass Mating

Hybridization was carried out as stated by [11], with some modifications. Spore suspensions were collected, mixed, and transferred into 25 mL of YPD broth and incubated for 3 days at 28 °C. Then, 200 µL of the culture was spread onto the selective agar plates until colony growth was observed. Hybrids of UCA-Y-001 × CLI-S were selected on the specific media previously determined based on complementary markers. In this case, it consisted of plates with YP-Melezitose agar and YP-Galactose agar. 

#### 2.3.3. Molecular Characterization

Genomic DNA extraction of yeast strains, both parental strains as well as putative hybrids, was performed using a fast extraction protocol proposed by [7]. Genomic DNA was used for PCR amplification with the inter-delta transposon primer set MLD1 5′-CAAAATTCACCTAAATTCTCA-3′ and MLD2 5′-GTGGATTTTTATTCCAACA-3′ [23]. PCR products were loaded on a 1.5% (*w*/*v*) agarose gel in TBE buffer 1X (40 mM Tris-acetate, 1 mM EDTA) and visualized by means of a UV transilluminator after staining with ethidium bromide 5 µg/mL. DNA fragment sizes were determined by comparison with a molecular ladder marker of 100 bp (GeneRuler 100 bp plus, Thermo Fisher, Waltham, MA, USA). 

### 2.4. Adaptive Laboratory Evolution 

To obtain low H_2_S-producing yeast solely from the parental strain UCA-Y-001, an evolution-based strategy previously described by [10] was applied, with some modifications.

In order to determine the concentration of molybdate with which the growth of the parental strain UCA-Y-001 was inhibited, a multi-plate test was carried out. Thus, YPD medium was supplemented with different concentrations of ammonium molybdate (50, 30, 20, 15, 12.5, 10, 7.5, 5, and 2.5 mM) and 180 µL of each medium was incorporated into the wells of polystyrene 96-well microplates (NuncTM 96-well polystyrene conical bottom MicrowellTM, ThermoFisher, Naerum, Denmark). Wells were inoculated with 20 µL of a yeast pre-culture (obtained in YPD medium after incubation in aerobic conditions at 28 °C for 12 h and then diluted to 0.1 OD 600 nm). Microplates were sealed with a Breathe-Easy membrane (Sigma, Saint Louis, MI, USA) and incubated at 28 °C over 48 h. Yeast growth in the absence of ammonium molybdate was used as a positive control.

After spore collection and dissection, cells were resuspended in 2 mL of YPD medium and incubated at room temperature until the conjugation of most of the spores was observed with an inverted microscope (AE2000, MoticEurope S.L.U., Barcelona, Spain). Then, 50 μL of suspension was inoculated in tubes containing 5 mL of YPD medium supplemented with the concentration of ammonium molybdate previously determined and incubated at 28 °C for 5 days. From each tube in which growth was observed, 100 μL were spread on YPD-ammonium molybdate plates and incubated at 28 °C for 5 days. From each plate in which growth was observed, a single colony was then reisolated on selective plates, purified and stored at −8 °C with glycerol 40%.

### 2.5. Qualitative Analysis of H_2_S Production

Hydrogen sulfide formation was qualitatively evaluated on bismuth-glucose-glycine-yeast (BiGGY) agar medium (Oxoid Co., Hampshire, UK) in triplicate as a first screening of the variants obtained in comparison with the parental strains. Ten μL of each strain was cultured in YPD medium until an OD_600_ of 1 was spotted on BiGGY agar and incubated for 5 days at 28 °C. On this differential medium, the colony colour turns dark, brown, or remains white depending on the amount of sulfide produced by the yeast [24].

### 2.6. Fermentation Trials

Laboratory scale fermentations were carried out in triplicate with the parental strain UCA-Y-001 and some of the evolved variants obtained in a filter-sterilized natural must of the Palomino Fino grape variety. An inoculum of 2 × 10^6^ cells/mL of each strain was added to 2-litre Erlenmeyer flasks containing 1 litre of natural must and kept static at 22 °C. Fermentations were monitored by daily weighing until a concentration of less than 2 g/L of residual sugar was reached. Flasks were sealed with rubber stoppers fitted with H_2_S-detecting silver nitrate tubes (120SF:1–1000 ppm; KITAGAWA, Fuchu, Hiroshima, Japan) as previously suggested [25].

Sherry base wines of the Palomino Fino grape variety were obtained under industrial-scale fermentation in a winery placed in the Sherry winemaking area (Jerez, Spain). They were performed in triplicate in stainless steel vessels of 400,000 l, and the inoculation with each selected yeast strain was carried out as previously described [6]. 

A pH-Meter Basic 20 (Crison Instruments, Barcelona, Spain) was used to measure the pH of the different wines. The alcoholic titer was analyzed by density measurement of the distillate in a DMA-5000 densimeter (Anton Paar, Ashland, OR, USA). Fermentative capacity and a velocity consumption of 100% of the sugar content (VF) as well as a fermentation velocity consumption of 50% of the sugar content (V50) were measured by daily weight loss checks during fermentation. On the other hand, titratable and volatile acidity, as well as free and total SO_2_, were determined by volumetry according to the official methods established by the International Organization of Vine and Wine (OIV).

### 2.7. Quantification of Carboxylic Acids and Volatile Compounds

Carboxylic acids were determined as stated in [7] by ionic chromatography using Dionex DX 500 equipment with a CD20 conductivity detector (Salt Lake City, UT, USA). Standard stock solutions of the organic acids (Sigma-Aldrich, St. Louis, MO, USA) were made by dissolution with distilled water. After filtering (0.22 µm) and dilution (1:20) with ultrapure water, white wine samples were injected into the chromatograph equipped with an IonPac ICE-AS6 capillary column. A concentration of 0.4 mM heptafluorobutyric acid (HFBA) (FlukaChemie AG, Buchs, Switzerland) was used as eluent at a flow rate of 1.0 mL/min in isocratic mode. An anion-Ice micro-membrane was used as suppressor column and tetrabutylammonium hydroxide (Sigma-Aldrich, St. Louis, MO, USA) as a regenerator with a flow rate of 5 mL/min. 

Volatile compound determinations (esters, alcohols, terpenes, norisoprenoids, and phenols) were carried out as stated [26], using SPE cartridges (LiChrolut^®^, 0.3 g of phase, Merck, Darmstadt, Germany) and 4-nonanol (0.1 g/L) as an internal standard. The extract was concentrated to a final volume of 150 μL by distillation in a Vigreux column and then under nitrogen stream and stored at –20 °C for further analysis. A Focus GC system coupled to a mass spectrometer ISQ with an electron-impact ionization source and a quadrupole analyzer equipped with an auto-sampler TriPlus (ThermoQuest, Waltham, MA, USA) was used to determine the free volatile composition of the different sherry base wines obtained at an industrial scale. Thus, a BP21 column (SGE, Ringwood, Australia) with a 60 m × 0.32 mm internal diameter and 0.25 µm thick Free Fatty Acid Phase (FFAP) was used. GC–MS conditions were the same as those described previously [27]. Volatile compounds were identified by chromatographic retention times and mass spectra, using commercial reagents as standards. Quantification was carried out by analyzing the trait m/z fragment for each compound using the internal standard method. Each wine was analyzed in triplicate.

### 2.8. Sensory Analysis

Sensory evaluations were performed under ISO standards related to methodology and sensory analysis vocabulary (ISO 8586:2014), selection and formation of tasters (ISO 11035:1994), and tasting room (ISO 8589:2007). Two different sensory analyses were performed in two sessions by eleven skilled adult judges (6 women and 5 men, mean age 29 years). The first round applied the Napping plus Ultra-Flash Profile (UFP), based on the classical Napping^®^ protocol [28]. All the wine samples (including the control) were presented to each assessor. The second sensory analysis was based on a hedonic test of the white wines with a second group of 30 (18 women and 12 men) regular white wine consumers with no formal wine training, as proposed in [29].

### 2.9. Statistical Analysis

The mean values of the different volatile compounds, as well as sensory analysis, were analyzed. To test the null hypothesis that the data sampled came from a normally distributed population, a Shapiro–Wilks test was applied. In other to determine the linear relationship among the different variables, Pearson’s correlation coefficient was also calculated. 

One-way ANOVA analysis was carried out to evaluate significant differences among wines made with the different yeast variants (significance level *p* < 0.05). Tukey’s test was used to highlight the effects of yeast variants compared with the parental yeast strain (significance level *p* ≤ 0.05). A principal component analysis (PCA) was also carried out.

Both the treatment and analysis of the data, as well as the plots, were carried out using programming in R software version 4.0.5 (R Core Team, 2020). All tests were performed in triplicate. 

## 3. Results

### 3.1. Selection of Lower H_2_S-Producing Yeast Variants

The sporulation capacity of both parental strains was firstly evaluated since hybridization can only take place between mating-competent cells (Table 1). Both strains showed a relatively acceptable sporulation efficiency; the parental strain UCA-Y-001 had a considerably lower capacity. Both parent yeast strains were diploid, heterozygous, and homothallic (HO/HO genotype). Therefore, it was expected that mass mating would produce gametes with loss of heterozygosity that could mat to give rise to intraspecific hybrids.

To distinguish putative hybrids UCA-Y-001 × CLI-S, complementary phenotypic markers were needed (Table S1). Regarding carbon source assimilation, the screening revealed that the parental yeast strain UCA-Y-001 was not able to grow on galactose, while the parental strain CLI-S could not grow in the presence of melezitose, using both as a sole carbon source in plates as selectable markers.

**Figure 1:**
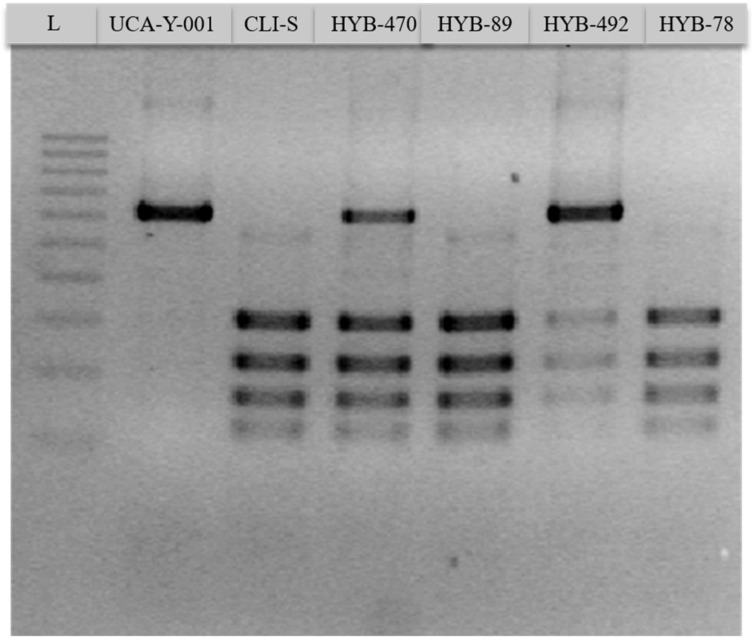
Transposon PCR of parental strains (UCA-Y-001, CLI-S) and some of the putative hybrids (HYB-470, HYB-89, HYB-492 and HYB-78). L: Generuler 100 bp plus.

Thus, after the selection of hybrids through the specific media, 18 potential hybrids were isolated then screened for H_2_S production on BiGGY-agar plates. All putative hybrids showed a significant reduction in H_2_S production according to this qualitative screening, as their colour was lighter compared to the parental UCA-Y-001 whose colour was dark brown. In addition, a transposon PCR was performed to confirm the mating products in the putative hybrids (Figure 1). Some of the putative hybrids showed a transposon PCR pattern intermediate between both parental strains, which displayed specific and different band patterns. This confirmed the hybrid nature of these.

**Figure 2:**
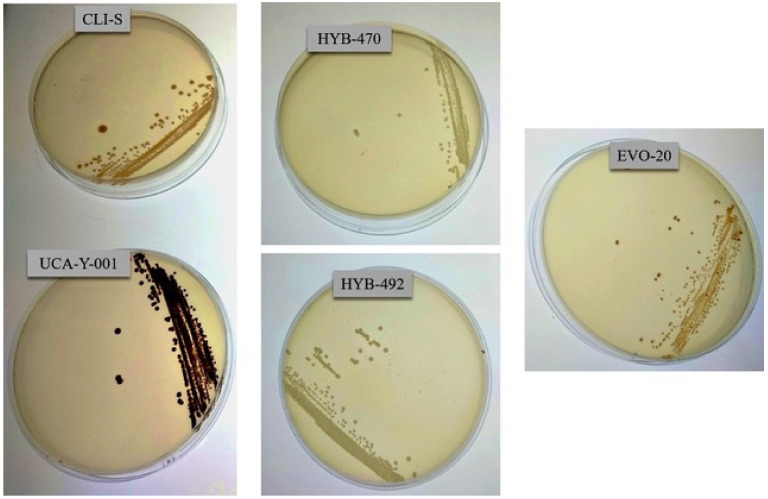
Qualitative evaluation of hydrogen sulfide on BiGGY agar plates of parental (UCA-Y-001, CLI-S) and the three evolved variant strains selected (HYB-470, HYB-492, EVO-20) to carry out fermentation at an industrial scale. The darker the colony, the more H_2_S is being produced.

Regarding adaptive laboratory evolution strategy, the parental yeast UCA-Y-001 was induced to sporulation and then, after sexual conjugation of spores, cell suspensions were inoculated in tubes containing YPD medium supplemented with 30 mM ammonium molybdate. After five days, growth was observed in fifteen of them, which were then spotted on selective medium plates; a total of 20 colonies were selected as candidates since they sowed a lighter colour than UCA-Y-001 on BiGGY agar plates, although in no case was a full white colour observed. Finally, three strains were selected as promising candidates (Figure 2): two hybrids (coded as HYB-470 and HYB-492), which displayed a full white colour, and an evolved variant obtained by adaptive laboratory evolution (coded as EVO-20).

Table 2 shows the principal oenological parameters of industrial Palomino Fino wines made with the parental yeast strain (UCA-Y-001), the evolved yeast strain (EVO-20), and the hybrid yeast strains (HYB-470 and HYB-492). The fermentative velocity (FV) of the evolved yeast strains was similar to the parental UCA-Y-001, although lower in the case of the EVO-20 yeast variant, which was also reflected in the alcoholic degree obtained in the different wines. 

Free SO_2_ was lower in evolved strains compared to the parental strain, coinciding with what was expected given the success of the two enhancement techniques employed. Furthermore, titratable acidity was also lower in wines obtained with evolved strains than in the control. Both hybrids (HYB-470 and HYB-492), especially, produced less acetic acid, while lactic acid was significantly lower in wines made with EVO-20.

### 3.2. Analytical Profiles of the Wines Obtained

Carboxylic acids and volatile compounds were analysed, having detected a total of 53 volatile compounds identified and quantified in the Palomino Fino white wines made with the parental yeast (UCA-Y-001) and the evolved strains (EVO-20, HYB-470, and HYB-492) (Table S2), which were also grouped according to their chemical structure (alcohols, esters, acids, carbonyl compounds, terpenes, norisoprenoids, lactones, thiols, and phenols). 

**Figure 3:**
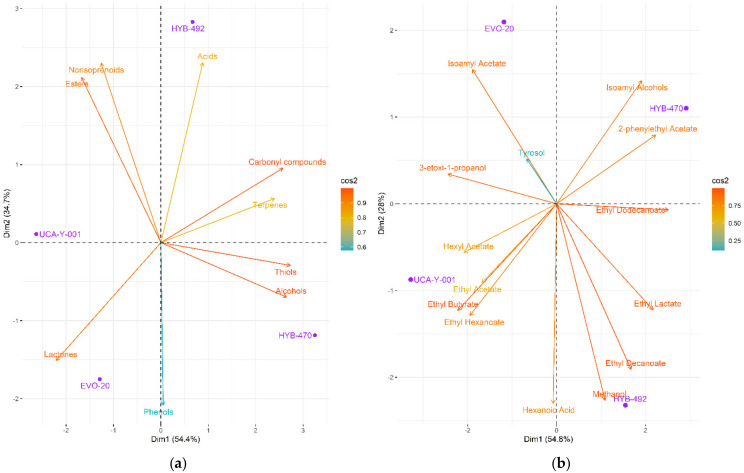
Principal component analysis of the volatile composition data of the industrial wines fermented with the different yeast strains studied (UCA-Y-001 as control, EVO-20, HYB-470, and HYB-492), using as variables: (**a**) chemical groups; (**b**) individual metabolites (on the plane, PC1 against PC2).

Correlations were found both for individual metabolites and chemical groups, obtaining their corresponding eigenvalues and eigenvectors. Variables between which the Pearson correlation was greater than 0.95 were determined, since they have a coefficient of determination greater than 90%, obtaining a total of fourteen metabolites. In general, differences between the four wines were observed. A PCA was carried out using the whole data to better appreciate similarities or differences among the wines analysed (Figure 3). The projection of both chemical groups (Figure 3a) and individual metabolites (Figure 3b) of the wine samples on the first two principal components (which explained 89.1% and 86.5% of total variability, respectively) showed that parental yeast and EVO-20 were differentiated from both hybrids (HYB-470 and HYB-492).

The main components responsible for the differences in both groups were alcohols/thiols (positively correlated with the PC1), esters, and terpenes (negatively correlated with the PC1). This agrees with what was expected considering the enhancement techniques implemented. In the case of adaptive evolution, the phenotypic variability of the parental yeast was exploited, while hybrids were obtained by crossing with another parental yeast which was considered interesting in this particular case (low acid and sulphite production). 

**Figure 4:**
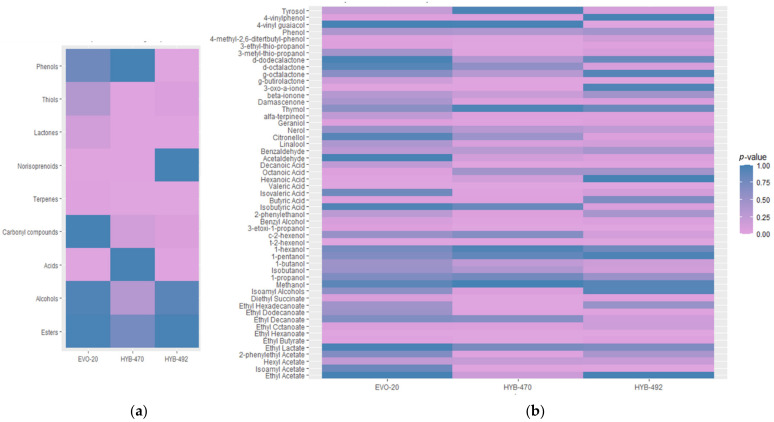
Heatmap representation of the significance of the volatile profile of the different wines obtained at an industrial scale compared to the control strain (UCA-Y-001): (**a**) chemical groups; (**b**) individual compounds. Significances were found according to ANOVA and Tukey’s test (*p*-value ≤ 0.05 is pale rose) compared with the control one. The palette turns blue when no differences were found between wines fermented with the evolved yeast strains and the control one.

Alcohols did not show significant differences between the wines analysed (Figure 4a), although a significant increase in 2-phenyl-ethanol (roses) and benzyl alcohol (fruity) was found in wines made with the HYB-470 variant, while there was an increase in the content of 4-vinyl guaiacol in the wine obtained by the HYB-492 variant (Figure 4b). On the other hand, t-2-hexenol (herbaceous) and 3-ethoxy-1-propanol (fruity) were significantly reduced in all wines compared to the control (Figure 4b). The 3-(ethylthio)-1-propanol content was also increased significantly in all wines made with evolved yeast strains.

Terpenes were significantly different in wines made with the evolved strains (Figure 4a). Specifically, α-terpineol showed a significant increase in the three different wines obtained with the three evolved yeasts compared to the control, mainly in both hybrids (HYB-470 and HYB-492). A significant increase in geraniol was also found, especially in wines made with the variant HYB-470 (Figure 4b).

Regarding the total content of esters, no significant differences were observed between the wines analysed (Figure 4a), although some significant differences have been found in some individual compounds (Figure 4b). This is the case with ethyl butyrate and ethyl hexanoate, which significantly decreased in the three wines compared to the control. On the other hand, ethyl dodecanoate and diethyl succinate significantly increased in the wines made with both hybrids (HYB-470 and HYB-492). 

### 3.3. Sensory Analysis

Wines fermented by the different yeast strains analysed (UCA-Y-001, EVO-20, HYB-470 and HYB-492) at an industrial level were evaluated by a total of eleven trained tasters, finally obtaining the scores attributed to the wines for twenty-four different descriptors and attributes. A correlation matrix was generated for them and used to analyze those among which the Pearson correlation was greater than 0.95; finally, eighteen descriptors were obtained that were found to have a correlation coefficient greater than 90%. 

**Figure 5:**
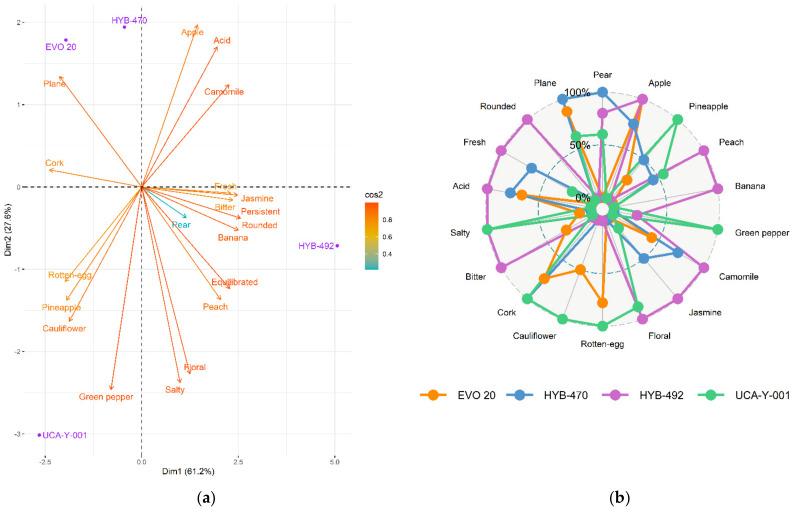
Sensory characterization of Palomino Fino white wines fermented with the different yeast strains studied (UCA-Y-001, EVO-20, HYB-470, and HYB-492) by Napping^®^: (**a**) PCA (on the plane, PC1 against PC2); (**b**) spider graph of the principal attributes in the obtained wines.

A PCA was performed (Figure 5a), thus showing that 88.8% of the variance was explained by the first two components. PC1 (61.2%) was positively correlated with white flowers on the nose, acidity, persistence, and aromatic complexity. On the other hand, it was negatively correlated with organoleptic defects, such as rotten egg, cauliflower, cork, and green pepper.

The white wine obtained through the parental strain (UCA-Y-001), which is currently used at an industrial level both for direct marketing and as a base wine in biological ageing, showed a dominance of tropical aromas (mainly peach) and a high presence of organoleptic defects, such as rotten egg, cauliflower, cork, or green pepper. On the palate, it was plain, salty, with low acidity and a light floral character (Figure 5b).

The wine obtained by adaptive evolution, EVO-20, was considered the most similar to the control wine, although it was more aromatic and fruity on the nose. The aroma of pear dominated, with hints of pineapple and notes of red flowers (roses). The presence of organoleptic defects was detected (cork, rotten egg, and ketones), although more faintly than in the control wine. It was described as having a balanced acidity and being medium-bodied in the mouth.

Wine elaborated with HYB-470 was considered to have a pleasant and fruity nose too, with aromas balanced between white fruits (pear) and tropical fruits (pineapple). Notes of white flowers stood out, such as chamomile and jasmine. However, the undesirable aroma of cork was present. In the mouth, it was described as fresh and simple.

Finally, the wine obtained with the HYB-492 variant was the most different according to the tasters. It was also described as a wine with a pleasant and fruity nose and with very balanced aromas between white fruits (pear and apple) and tropical fruits (banana, peach, and pineapple). Aromatic notes of white flowers (chamomile, jasmine, and orange blossom) also stood out. On the palate, it was well-defined, acidic, fresh, persistent and with a creamy mouthfeel.

## 4. Discussion

In the present study, to achieve an improvement of the wine quality that was already produced at an industrial level, it was necessary to previously obtain evolved variants of the parental yeast strain (UCA-Y-001) relative to the sulphate assimilation pathway with subsequent lower production of both SO_2_ and H_2_S. For this purpose, two different enhancement strategies were carried out: adaptive laboratory evolution of the parental strain, thus exploiting its natural diversity, and hybridization of the said industrial selected yeast strain with another which was also previously selected in another low aromatic grape variety (named CLI-S). 

Regarding the sporulation efficiency, the low capacity recorded by the two parental strains was not surprising, since industrial yeasts often present polyploidy and display a low sporulation efficiency and low spore viability [30].

It is well known that wine yeasts, especially those belonging to the species *S. cerevisiae*, have technical advantages of sexual recombination that can be exploited to generate a genetic variant library of a specific strain [12]. In this way, sexual hybridization techniques are the most efficient way to generate artificial diversity in yeasts [31]. However, when the aim is to improve a certain industrial yeast strain that has already been selected in a given environment for some specific oenological traits, as in this case, hybridization, either inter- or intraspecies, can introduce greater phenotypic variability that may extend far beyond the initial purpose. For this reason, an adaptive laboratory evolution was also employed. It was achieved by exploiting the phenotypic variability of the parental strain and applying a strong selective pressure for the selection of the phenotypes of interest. In this way, resistance to toxic analogues of sulphate was proposed as a high-throughput and rapid screening method for obtaining evolved strains with lower production of sulphites and H_2_S due to an inability to assimilate sulphates, since high-affinity sulphide permeases remained inactive [10,32].

In total, 10 yeast variants (2 hybrids and 8 evolved variants obtained by adaptive evolution) complied with the oenological parameters studied and, consequently, presented phenotypic characteristics closer to their parental yeast UCA-Y-001. They were used to carry out fermentation in synthetic must and natural must to select those that could be of interest for further analysis at the industrial level (data not shown). Sensory analysis was developed by a trained tasting panel and winery members by using the quick profile and nap method (data not shown). Finally, three strains were selected as promising candidates to carry out fermentation at an industrial level: two hybrids (coded as HYB-470 and HYB-492) and one strain obtained by adaptive laboratory evolution (coded as EVO-20).

After analysing the wines obtained, alcohols did not show significant differences between the wines analysed (Figure 4a), which was desirable, as C6 alcohols are proposed as a potential marker of varietal authenticity [33] and some authors have even described them as major contributors to the varietal aroma of neutral grape varieties [2,34]. Although vinyl phenols can be responsible for heavy pharmaceutical odours in white wines at high concentrations [35,36], in lower concentrations, as in this case, they are related to a pleasant spicy or herbaceous aroma. Specifically, 4-vinyl guaiacol imparts interesting notes to some white wines, since it can give a clove-like aroma, and is the main compound responsible for the spicy aroma of Gewürztraminer’s wines [37]. It is also interesting to note that all the wines obtained with the evolved variants showed a significant increase in 3-(ethylthio)-1-propanol, whose formation is related to the catabolic pathway of methionine [38].

On the other hand, terpenes are considered the main part of the varietal compounds derived from grapes. They are usually glycosidically bound [39], depending on the variety and the relative proportions of free and bound forms [16]. Terpene glycosides are hydrolysed to free volatile terpenes by yeast glycosidase during fermentation [2], thus providing floral aromatic notes in wines made with all three evolved stains, as was especially the case with both hybrids.

Glycerol and 2,3-butanediol are not compounds that properly influence the aroma of wines, but they play an essential role in their viscosity and mouthfeel [7]. Although there was a slight decrease in these polyalcohols in wines made with the evolved variants, differences were not significant—a desirable result, since, in this particular case, they play an even more important role in being aerobically assimilated by the yeast flor during biological ageing of the base wine [40]. In addition, it should be noted that glycerol formation during wine fermentation can also be affected by nitrogen availability for yeasts [41] in the same way that the production of hydrogen sulfide is influenced [42].

Even though the base wine for biological ageing is obtained from a low aromatic grape variety, if the quality and complexity of the wine are increased, not only will the quality of the wines that undergo biological ageing be directly influenced but also the young white wine could be directly commercialized.

In general, wines made with the evolved strains showed the highest scores for most desirable attributes, especially aromatic complexity and a fruity nose, thus obtaining a noticeable improvement over the wine made with the control yeast (UCA-Y-001). Furthermore, organoleptic defects were diminished when evolved yeasts were used, especially in the case of both hybrids (Figure 5a). According to the analysis of the different wines obtained, the most similar wine to that produced by the parental strain was obtained through the EVO-20 variant, while the most different was the wine obtained with the HYB-470 variant. 

Thus, wines with greater organoleptic complexity were obtained from a parental yeast strain that was already used to produce young white wine at an industrial level. The results were achieved through the use of two techniques that exploit yeast genetic variability, obtaining three different wines at an industrial level quite similar to that of the parent strain. Chemical data confirmed the existence of significant differences between the wines made with the different variants. In addition, those differences were also detected at the sensory level, the wines being generally more appreciated than the control wine by tasters. 

## Figures and Tables

**Table 1:** Mating type, sporulation efficiency, and spore viability of the parental yeast strains (UCA-Y-001 and CLI-S).

Strain	Mating Type	Time Asci Formation (Days)			Viability (%)	Efficiency (%)
		3	7	15		
UCA-Y-001	MATa/MATα HO/HO	−	+	+	27.3	37
CLI-S	MATa/MATα HO/HO	+	+	+	57	79

**Table 2:** Oenological parameters (mean value ± standard deviation, *n* = 3) of Palomino Fino white wines fermented with the different yeast strains analyzed at an industrial level.

	UCA-Y-001	EVO-20	HYB-470	HYB-492
Fermentative capacity	13.00 ± 0.04 ^d^	12.00 ± 0.02 ^a^	12.76 ± 0.03 ^c^	12.71 ± 0.02 ^c^
V_50_	20.30 ± 0.06 ^d^	18.40 ± 0.04 ^b^	19.83 ± 0.02 ^c^	19.78 ± 0.03 ^c^
V_F_	7.72 ± 0.02 ^d^	6.30 ± 0.01 ^c^	6.50 ± 0.00 ^d^	6.00 ± 0.01 ^b^
Alcoholic degree ^1^	12.91 ± 0.01 ^d^	11.80 ± 0.01 ^a^	12.82 ± 0.02 ^c^	12.76 ± 0.02 ^b^
Titratable acidity ^2^	5.00 ± 0.03 ^c^	3.97 ± 0.20 ^a^	4.61 ± 0.01 ^b^	4.78 ± 0.11 ^bc^
pH	3.54 ± 0.02 ^c^	3.48 ± 0.02 ^b^	3.63 ± 0.00 ^d^	3.61 ± 0.00 ^d^
Free SO_2_ ^3^	38 ± 0 ^d^	25 ± 0 ^c^	21 ± 0 ^b^	20 ± 0 ^a^
Total SO_2_ ^3^	168 ± 1 ^c^	177 ± 3 ^d^	154 ± 4 ^b^	151 ± 1 ^ab^
Reducing sugar ^4^	0.60 ± 0.20 ^b^	0.55 ± 0.02 ^b^	1.00 ± 0.02 ^c^	0.19 ± 0.00 ^a^
Acetic acid ^4^	0.23 ± 0.02 ^b^	0.23 ± 0.01 ^b^	0.15 ± 0.00 ^a^	0.12 ± 0.02 ^a^
Citric acid ^4^	0.27 ± 0.00	0.25 ± 0.01	0.27 ± 0.00	0.26 ± 0.01
Malic acid ^4^	1.66 ± 0.01 ^b^	1.54 ± 0.04 ^a^	1.67 ± 0.04 ^b^	1.66 ± 0.03 ^b^
Lactic acid ^4^	0.18 ± 0.01 ^b^	0.09 ± 0.00 ^a^	0.18 ± 0.02 ^b^	0.20 ± 0.01 ^bc^
Succinic acid ^4^	0.24 ± 0.06	0.23 ± 0.02	0.29 ± 0.03	0.30 ± 0.10
Glycerol ^4^	6.21 ± 0.17 ^b^	5.45 ± 0.03 ^a^	5.52 ± 0.04 ^a^	5.74 ± 0.24 ^a^
2.3-butanediol ^3^	246 ± 31 ^a^	200 ± 56 ^a^	221 ± 49 ^a^	198 ± 41 ^a^

V_50_ = fermentation velocity consumption of 50% of the sugar content; V_F_ = fermentation velocity (% of daily sugar consumption). ^1^ %, *v*/*v*. ^2^ Tartaric acid, g/L. ^3^ mg/L. ^4^ g/L. The characters a, b, c, d mean significant differences between for yeast strain variants studied (*p* ≤ 0.05).

## Data Availability

Not applicable.

## Supplementary Materials

The following supporting information can be downloaded at: https://www.mdpi.com/article/10.3390/foods11081104/s1, Table S1: Phenotypic characteristics of parental yeast strains (UCA-Y-001 and CLI-S), Table S2: Concentration of volatile compounds in Palomino Fino white wines fermented with the different yeast strains used in this study (UCA-Y-001 as control; EVO-20, HYB-470, and HYB-492).
