# Supplementary material for: Improving an Industrial Sherry Base Wine by Yeast Enhancement Strategies
Source: Foods. 2022 Apr 12;11(8):1104. doi: 10.3390/foods11081104 (PMC9030371; doi:10.3390/foods11081104)
Supplement: Supplementary file 1 [file foods-11-01104-s001.zip › foods-1660467-supplementary.pdf]

foods

MDPI

Supplementary Materials

Improving an Industrial Sherry Base Wine by Yeast Enhancement Strategies

Marina Ruiz-Muñoz [1], Gustavo Cordero-Bueso [1,*], Pedro Miguel Izquierdo-Cañas [2,3], Adela Mena-Morales [2] and Jesús M. Cantoral [1]

1 Departamento de Biomedicina, Biotecnología y Salud Pública, Universidad de Cádiz, Avenida República Árabe Saharaui s/n, Puerto Real, 11510 Cadiz, Spain; marina.ruiz@uca.es (M.R.-M.); jesusmanuel.cantoral@uca.es (J.M.C.)
2 Instituto Regional de Investigación y Desarrollo Agroalimentario y Forestal de Castilla-La Mancha (IRIAF), IVICAM. Ctra. Albacete s/n, Tomelloso, 13700 Ciudad Real, Spain; pmizquierdo@jccm.es (P.M.I.-C.); amenam@jccm.es (A.M.-M.)
3 Parque Científico y Tecnológico de Castilla-La Mancha, Paseo de la Innovación 1, 02006 Albacete, Spain
* Correspondence: gustavo.cordero@uca.es

Table S1. Phenotypic characteristics of parental yeast strains (UCA-Y-001 and CLI-S).

Fermentation	UCA-Y-001	CLI-S
D-Glucose	+	+
D-Galactose	-/w	+
Lactose	w	-
Maltose	+	+
Sucrose	+	+
Melibiose	+	+
Raffinose	+	+
Trehalose	w	-
**Assimilation**	**UCA-Y-001**	**CLI-S**
D-Glucose	+	+
D-Galactose	-	+
Lactose	-	-
Maltose	+	+
Sucrose	+	+
Melibiose	-	-
Raffinose	+	+
Inulin	+	+
Cellobiose	-	-
Trehalose	w	-
Melezitose	+	-
Methil-α-D-Glucoside	-	-
Soluble Starch	-	-
Salicin	-	-
L-Rhamnose	-	-
L-Sorbose	w	-
D-Xylose	w	-
L-Arabinose	-	-
D-Arabinose	-	-
D-Ribose	w	-
Methanol	-	-
Ethanol 8%	+	+
Ethanol 10%	+	+
Ethanol 11%	-	+
Ethanol 14%	-	w

Glycerol	-	-
Erythritol	-	w
Cadaverine	-	-
Ethylamine	+/w	-
Galactitol	-	-
D-Manitol	-	-
D-Glucitol	-	-
Myo-inositol	-	-
D-L-Lactate	-	-
Succinate	-	-
Citrate	-	-
D-Gluconate	-	+/w
D-Glucosamine	+	-
N-Acetyl Glucosamine	-	-
Hexadecane	-	-
L-Lysine	-	-
Nitrate	+	+
Nitrite	w	w
2-Keto-D-gluconate	-/w	-
L-Arabinitol	-	-
Arbutine	-	-
Propane 1,2-diol	-	-
Butane 2,3-diol	-	-
Creatinine	+	-
Urease	+/w	-
**Growth**	**UCA-Y-001**	**CLI-S**
Cycloheximide 1000 ppm	-	-
Cycloheximide 100 ppm	-	-
50% Glucose	-	-
10% NaCl	+	-
16% NaCl	-	-
pH 3	+	+
pH 9.5	-	+
YM broth at 25ºC, 3 days	+	+
YPD broth at 28ºC, 3 days	+	+
YPD broth at 37ºC, 3 days	-	+
YPD broth at 17ºC, 3 days	+	+
YPD broth at 13ºC, 3 days	+	-
Cellular reproduction	Asymmetrically by budding	Asymmetrically by budding
Pseudohyphae or true hyphae formation	Not Formed	Not Formed
Sporulation	Yes (2:2)	Yes (2:4)
H_2S/L (µg)	3.06	0.04
Biggy agar phenotype	Dark brown	white
Killer phenotype (K)	K-positive	K-positive
Fermentative capacity	12.9	12.8
Volatile acidity(g/L; acetic acid)	0.7	0.4
Titratable acidity (g/L; tartaric acid)	1.89	2.75
Citric acid	nd	0.31
Malic acid	nd	2.82
Lactic acid	nd	0.59
Acetic acid	nd	0.17
Succinic acid	nd	0.29
Glycerol (g/L)	8.6	7
2.3-butanodiol (mg/L)	441	373
Killer phenotype (K)	K-positive	K-positive
Acetaldehyde (mg/L)	nd	50.75
Acetoin (mg/L)	nd	tr

Ethyl acetate (mg/L)	nd	68.71
Higher major alcohols (mg/L):		
1-Propanol	nd	35.02
Isobutanol	nd	37.39
Isoamylic alcohols	nd	175.23
1-Hexanol	nd	0.81
2-Phenylethanol	nd	10.79
Isobutyl acetate	nd	0.16
Isoamyl acetate	nd	7.66
Hexyl acetate	nd	0.14
Phenyl ethyl acetate	nd	0.56
Ethyl butyrate	nd	0.33
Ethyl hexanoate	nd	0.60
Ethyl octanoate	nd	1.01
Ethyl decanoate	nd	0.08
Ethyl lactate (mg/L)	nd	tr
Diethyl succinate (mg/L)	nd	0.03
SCFA (mg/L):		
Isobutyric acid	nd	3.01
Butyric acid	nd	1.90
Isovaleric acid	nd	0.08
Hexanoic acid	nd	4.97
Octanoic acid	nd	6.11
Decanoic acid	nd	3.70
4-vinylguaiacol	nd	0.47

Table S2. Concentration of volatile compounds in Palomino Fino white wines fermented with the different yeast strains used in this study (UCA-Y-001 as control; EVO-20, HYB-470 and HYB-492).

	UCA-Y-001	EVO-20	HYB-470	HYB-492
Ethyl Acetate [1]	65.98 ± 0.65	66.84 ± 3.40	54.35 ± 5.40	66.84 ± 6.14
Isoamyl Acetate [1]	4.51 ± 0.05	4.30 ± 0.30	2.97 ± 0.02	1.27 ± 0.02
Hexyl Acetate [2]	35.23 ± 8.20	22.60 ± 4.82	21.90 ± 1.97	21.28 ± 4.03
2-phenylethyl Acetate [2]	186.65 ± 17.42	252.50 ± 30.06	296.11 ± 5.97	286.33 ± 91.81
Ethyl Lactate [1]	19.59 ± 0.93	17.84 ± 0.89	25.45 ± 3.33	25.77 ± 10.56
Ethyl Butyrate [2]	411.00 ± 25.46	281.50 ± 4.95	174.50 ± 4.24	302.00 ± 35.36
Ethyl Hexanoate [1]	1.69 ± 0.08	0.71 ± 0.03	0.64 ± 0.07	0.93 ± 0.13
Ethyl Octanoate [1]	2.00 ± 0.43	1.09 ± 0.03	1.05 ± 0.11	1.46 ± 0.20
Ethyl Decanoate [2]	385.49 ± 27.56	312.26±105.27	464.02 ± 29.73	564.51 ± 46.12
Ethyl Dodecanoate [2]	18.02 ± 1.59	27.28 ± 5.41	62.47 ± 1.15	48.87 ± 8.52
Ethyl Hexadecanoate [2]	20.12 ± 3.28	13.19 ± 4.02	55.77 ± 6.97	26.46 ± 6.35
Diethyl Succinate [2]	70.43 ± 3.97	119.22 ± 9.94	216.56 ± 20.49	134.21 ± 24.52
**Esters [1]**	**94.90 ± 1.26**	**91.81 ± 4.45**	**85.96 ± 3.46**	**97.55 ± 16.68**
Isoamyl Alcohols [1]	174.47 ± 1.36	196.11 ± 12.69	236.83 ± 8.08	184.44 ± 30.36
Methanol [1]	68.84 ± 4.19	61.26 ± 2.99	69.34 ± 3.84	75.88 ± 20.33
1-propanol [1]	62.96 ± 2.60	56.08 ± 3.02	56.86 ± 1.97	53.73 ± 11.25
Isobutanol [1]	27.97 ± 0.28	35.01 ± 1.99	36.84 ± 2.86	41.64 ± 8.95
1-butanol [1]	0.90 ± 0.07	1.23 ± 0.03	1.38 ± 0.29	1.49 ± 0.32
1-pentanol [2]	30.79 ± 2.94	24.65 ± 6.30	26.68 ± 2.83	28.35 ± 6.40
1-hexanol [1]	0.95 ± 0.07	1.09 ± 0.13	1.02 ± 0.09	1.09 ± 0.22
t-2-hexenol	4.92 ± 0.97	0.77 ± 0.23	1.37 ± 0.24	0.45 ± 0.17
c-2-hexenol	4.79 ± 1.12	6.31 ± 1.56	6.05 ± 0.38	7.97 ± 0.54
3-etoxi-1-propanol	79.08 ± 7.03	53.10 ± 8.61	24.72 ± 3.83	20.44 ± 2.97
Benzyl Alcohol	46.82 ± 4.21	67.91 ± 7.35	80.22 ± 5.71	70.41 ± 6.92
2-phenylethanol [1]	9.24 ± 0.93	13.56 ± 3.33	17.66 ± 4.14	12.71 ± 1.85
**Alcohols [1]**	**345.50 ± 6.94**	**364.51 ± 17.27**	**420.08 ± 7.10**	**371.10 ± 7.82**
Isobutyric Acid	191.02 ± 19.99	168.26 ± 35.32	229.625 ± 49.43	351.80 ± 68.87
Butyric Acid [1]	1.56 ± 0.12	0.76 ± 0.04	0.69 ± 0.10	1.35 ± 0.35
Isovaleric Acid [1]	0.40 ± 0.11	0.33 ± 0.05	1.06 ± 0.09	0.64 ± 0.05
Valeric Acid	50.52 ± 4.81	5.82 ± 0.67	10.81 ± 1.89	12.18 ± 3.12
Hexanoic Acid [1]	19.28 ± 1.03	9.33 ± 0.78	14.96 ± 0.34	19.05 ± 2.53
Octanoic Acid [1]	28.55 ± 2.59	19.90 ± 2.02	25.17 ± 0.37	31.94 ± 2.25
Decanoic Acid [1]	3.26 ± 0.98	5.91 ± 0.28	11.09 ± 0.62	9.00 ± 1.55
**Acids [1]**	**53.30 ± 0.37**	**36.40 ± 1.57**	**52.94 ± 0.62**	**62.35 ± 1.60**
Acetaldehyde [1]	15.58 ± 2.20	18.01 ± 0.06	50.11 ± 0.12	60.06 ± 21.08
Benzaldehyde [2]	7.84 ± 1.94	5.12 ± 0.76	5.23 ± 0.79	5.64 ± 0.50
**Cabonyl Compounds [1]**	**15.59 ± 2.20**	**18.01 ± 0.06**	**50.13 ± 0.12**	**45.07 ± 21.08**
Linalool [2]	8.33 ± 1.04	11.83 ± 0.78	14.79 ± 3.61	14.07 ± 3.37
Citronellol [2]	1.03 ± 0.25	1.50 ± 0.41	2.16 ± 1.11	3.96 ± 1.30
Nerol [2]	0.31 ± 0.16	0.63 ± 0.31	0.75 ± 0.10	0.81 ± 0.16
Geraniol [2]	3.29 ± 0.84	7.47 ± 1.47	8.42 ± 1.44	7.40 ± 1.00
alfa-terpineol [2]	4.23 ± 0.47	6.07 ± 0.69	7.95 ± 0.91	7.91 ± 0.99
Thymol [2]	1.50 ± 0.53	1.01 ± 0.41	1.31 ± 0.23	1.16 ± 0.20
**Terpenes [2]**	**18.67 ± 0.91**	**28.51 ± 2.64**	**35.39 ± 3.17**	**35.30 ± 2.03**
Damascenone [2]	2.96 ± 0.80	4.38 ± 0.57	6.45 ± 0.72	5.95 ± 1.03
beta-ionone [2]	0.06 ± 0.02	0.11 ± 0.01	0.13 ± 0.03	0.10 ± 0.00
3-oxo-a-ionol [2]	62.31 ± 4.25	34.51 ± 4.23	30.94 ± 0.77	60.10 ± 3.50
**Norisoprenoids [2]**	**65.32 ± 3.43**	**39.00 ± 4.79**	**37.52 ± 1.46**	**66.15 ± 2.47**
g-butirolactone [1]	0.81 ± 0.04	0.98 ± 0.08	0.38 ± 0.03	0.38 ± 0.05
g-octalactone [2]	1.18 ± 0.26	0.82 ± 0.25	1.74 ± 0.18	1.01 ± 0.21
d-octalactone [2]	22.94 ± 2.54	21.05 ± 3.17	18.72 ± 3.30	12.58 ± 4.62
d-dodecalactone [2]	26.68 ± 6.27	28.74 ± 7.75	38.28 ± 4.80	31.90 ± 5.56
**Lactones [1]**	**0.86 ± 0.04**	**1.03 ± 0.07**	**0.44 ± 0.03**	**0.43 ± 0.06**
3-methyl-thio-propanol [2]	58.64 ± 4.54	103.09 ± 5.49	237.28 ± 11.06	146.16 ± 35.38

3-ethyl-thio-propanol [2]	1.03 ± 0.39	7.72 ± 0.59	18.69 ± 0.32	8.37 ± 0.94
**Thioalcohols [2]**	**59.67 ± 4.93**	**110.81 ± 6.08**	**255.97 ± 10.74**	**154.52 ± 36.32**
4-methyl-2,6-ditercbutil-phenol [2]	48.31 ± 4.54	22.16 ± 2.99	83.43 ± 4.30	31.99 ± 7.36
Phenol [2]	2.82 ± 1.00	1.50 ± 0.42	1.45 ± 0.65	1.62 ± 0.59
4-vinyl guaiacol [1]	0.49 ± 0.13	0.56 ± 0.08	1.05 ± 0.08	19.23 ± 2.62
4-vinyl phenol [2]	7.21 ± 1.67	1.89 ± 0.53	2.16 ± 0.36	7.24 ± 1.74
Tyrosol [1]	4.75 ± 0.34	3.60 ± 0.17	5.57 ± 0.77	3.24 ± 0.48
**Phenols [1]**	**5.30 ± 0.48**	**4.19 ± 0.10**	**5.54 ± 0.69**	**2.25 ± 2.13**

Mean values ± standard deviation expressed as [1] mg/l or [2] µg/l.
